# Letter on “(1,3)-β-d-Glucan-based empirical antifungal interruption in suspected invasive candidiasis: a randomized trial”

**DOI:** 10.1186/s13054-020-03450-z

**Published:** 2021-02-09

**Authors:** Antonios Kritikos, Frederic Lamoth

**Affiliations:** Microbiology Institute and Infectious Diseases Service, Lausanne University Hospital (CHUV), Rue du Bugnon 46, 1011 Lausanne, Switzerland

**To the Editor:** *De Pascale *et al. recently reported on the potential role of (1,3)-β-d-glucan (BDG)-guided strategy as an antifungal stewardship tool in patients with sepsis/septic shock and risk factors for invasive candidiasis (IC) [1]. This raises the question of the possible drawback of false-positive results, which may lead to antifungal treatment (AF) overconsumption.

Empirical AF in intensive care unit (ICU) is current practice, although a recent randomized placebo-controlled trial did not demonstrate any survival benefit [2]. While the Infectious Diseases Society of America provides recommendations on stopping empirical AF in case of a negative non-culture-based assay (e.g. BDG) [3], the management of cases with positive BDG and no further evidence of IC remains a matter of debate.

Authors argue that the duration of therapy (median 8 days) in such patients was the same as of controls. The open-label design of the study is nonetheless concerning. A positive initial BDG test, of which clinicians were aware of, was present in most patients of the control group and could have led to a bias by extending treatment duration in this group.

The specificity and positive predictive value (PPV) of BDG in ICU vary across studies. The diagnostic criteria of IC, the proportion of candidemia versus non-candidemic IC, and most importantly, the targeted population of BDG testing and prevalence of the disease could influence diagnostic performances. In the present study, the PPV of BDG for IC diagnosis was 37% for a prevalence of IC of 12% [1]. We observed comparable results in a study reporting our experience of BDG testing in ICU (PPV 36%, IC prevalence 19%) [4]. This means that two out of three patients could receive unnecessary AF based on a positive BDG result. PPV was considerably higher (70–80%) among high-risk patients with complicated abdominal surgery [4, 5]. However, from our experience, only 26% of BDG tests were performed in an appropriate setting in real-life ICU conditions and BDG results were not considered in therapeutic decisions in 43% of cases [4]. In Table 1, we compare the pretest and posttest probability of IC in case of positive BDG, and we try to delineate the role of BDG testing in three risk categories.

**Table 1:** Empirical antifungal therapy initiated for patients with severe sepsis despite broad-spectrum antibiotics or septic shock: management of positive BDG results in the absence of formal IC documentation

Clinical setting	IC prevalence (pretest probability)	BDG PPV^b^ (posttest probability)	Role/impact of BDG^c^	Management of positive BDG results^d^
CS < 3/CCI < 0.5	< 10%	< 20%	No	Consider stop AF if negative cultures (whatever BDG results)
CS ≥ 3/CCI ≥ 0.5	10–20%	20–40%	Moderate	Consider stop AF or short AF therapy (5–7 days) if negative cultures and no suspected/documented uncontrolled source of infection Consider AF continuation (treat-like IC) in specific situations, e.g. suspected/documented uncontrolled source of infection and no culture available
Complicated abdominal surgery^a^	30–40%	50–70%	Yes	Consider AF continuation (treat-like IC)

^a^(i) Anastomotic leakage, (ii) recurrent gastrointestinal perforation or severe necrotising pancreatitis, (iii) recent abdominal surgery (< 7 days) and total parenteral nutrition and ongoing broad-spectrum antibiotic [4, 5]

^b^BDG PPV calculated according to IC prevalence for a specificity of 70–80%

^c^Assessment of the role of BDG takes into consideration a turnaround time for BDG results of 2–3 days (similar to culture in real laboratory workflow conditions)

^d^The negative predictive value of BDG is considered as > 90% in all settings, and AF interruption should be considered in all cases if negative BDG

Implementation of BDG testing in ICU may be beneficial if integrated in antifungal stewardship strategies including testing indications/interpretation of results and constant monitoring of practices. Studies assessing the overall impact and cost-effectiveness of BDG testing in ICU are needed.

## Data Availability

Not applicable.

## Abbreviations

BDG: (1,3)-β-d-Glucan
IC: Invasive candidiasis
AF: Antifungal treatment
ICU: Intensive care unit
IAC: Intra-abdominal candidiasis
PPV: Positive predictive value
