# Supplementary material for: Letter on “(1,3)-β-d-Glucan-based empirical antifungal interruption in suspected invasive candidiasis: a randomized trial”
Source: Crit Care. 2021 Feb 9;25:55. doi: 10.1186/s13054-020-03450-z (PMC7874441; doi:10.1186/s13054-020-03450-z)
Supplement: Related texts

# Reply: Authors’ response

**Authors:** Salvatore Lucio Cutuli, Gennaro De Pascale, Domenico Luca Grieco, Maurizio Sanguinetti, Brunella Posteraro, Massimo Antonelli

**Affiliations:** 2 grid.411075.6 0000 0004 1760 4193 Dipartimento di Scienze dell’Emergenza, Anestesiologiche e della Rianimazione, Fondazione Policlinico Universitario Agostino Gemelli IRCCS, Largo A. Gemelli 8, 00168 Rome, Italy; 3 grid.8142.f 0000 0001 0941 3192 Istituto di Anestesia e Rianimazione, Università Cattolica del Sacro Cuore, Largo F. Vito 1, 00168 Rome, Italy; 4 grid.8142.f 0000 0001 0941 3192 Istituto di Microbiologia, Università Cattolica del Sacro Cuore, Largo F. Vito 1, 00168 Rome, Italy; 5 grid.414603.4 Dipartimento di Scienze di Laboratorio e Infettivologiche, UOC di Microbiologia, Fondazione Policlinico Universitario A. Gemelli IRCCS, Largo A. Gemelli 8, 00168 Rome, Italy; 6 grid.414603.4 Dipartimento Di Scienze Gastroenterologiche, Endocrino-Metaboliche e Nefro-Urologiche, Fondazione Policlinico Universitario A. Gemelli IRCCS, Largo A. Gemelli 8, 00168 Rome, Italy; 7 grid.8142.f 0000 0001 0941 3192 Istituto di Patologia Medica e Semeiotica Medica, Università Cattolica del Sacro Cuore, Largo F. Vito 1, 00168 Rome, Italy; 8 grid.8142.f 0000 0001 0941 3192 Fondazione Policlinico A. Gemelli IRCCS - Università Cattolica del Sacro Cuore, Largo A. Gemelli 8, 00168 Rome, Italy

To the Editor,

the concerns of Drs Kritikos and Lamoth [6], raised by the risk of inappropriate exposure to empirical antifungal therapy in patients with suspected invasive candidiasis (IC), are well known to the scientific community and have been discussed in detail by recent papers [7, 8]. In the light of this view, we designed a randomized controlled trial [1] and verified the hypothesis that (1,3)-β-D glucan (BDG)-based strategy efficiently limits the exposure of critically ill patients at high risk of IC to inappropriate antifungal therapy. However, we are fully aware that pretest likelihood of IC may impact on BDG results [8], and such concept should be considered by the physician in order to prevent therapeutic choices that, otherwise, may appear de-personalised and not oriented to patient’s characteristics. Accordingly, this concept represents the most clinically sound explanation of the duration of empirical antifungal therapy among controls, in which 45.5% of participants were surgical patients, that are known to be a population at significant risk of IC [7]. For this reason, they received empirical antifungal therapy until the suspicion of IC was denied by culture-based test, regardless of baseline BDG. Uneventfully, in our trial, false-positive BDG results implied a per-protocol duration of empirical antifungal therapy until it was below 80 pg/ml, which could have been even shorter when considering the pretest likelihood of IC in a daily clinical frame. Although Drs Kritikos and Lamoth [6] argued against the use of antifungals in unconfirmed (thus suspected) IC, we would like to remind that the burden of time-limited exposure to such therapy may be considered negligible compared to the lethality of delayed treatments when IC occurs [7]. To conclude, BDG-based strategy reduces the risk of inappropriate exposure to empiric antifungal therapy in severe critically ill patients with suspected ICI. Moreover, BDG clinical performance may be even improved by concurrent and integrated use of biomarkers (e.g. procalcitonin, mannan and anti-mannan serum assays [1, 7, 8]), in order to provide a prompt and objective diagnosis of IC, thus orienting the clinical management of this life-threatening clinical condition.
